# James George Davey

**Published:** 1895-03

**Authors:** 


### Obituary.

---

### JAMES GEORGE DAVEY,

### M.D., M.R.C.S., L.R.C.P., L.S.A.

We have once more to record the death of one of the former Presidents of our Society.

Dr. J. G. Davey died on February 20th, at the advanced age of 82. He began his professional life as a general practitioner, but circumstances soon transferred him to the special branch of medical work which he continued to the end, viz., the treatment of lunacy and the care of the insane; and it will be seen from this short account of his life that in these he had a very large and varied experience.

James George Davey was born in 1812; he became L.S.A. in 1833, M.R.C.S. in 1836, L.R.C.P. in 1842 and M.D. of St. Andrew's. He started in general practice at Portsmouth in 1835, but about 1840 he succeeded in obtaining an appointment as one of the Medical Officers of Hanwell Lunatic Asylum, which he held until 1844. So well did he satisfy the authorities that he was selected to organise and superintend an asylum that was being built at Colombo, Ceylon, for the reception of Cingalese and other native lunatics, who had hitherto been housed with lepers and others of the natives afflicted with maladies requiring seclusion or confinement. He carried on the work at Ceylon for six years, when, chiefly on economical grounds, a change was made and the duties were transferred to one of the army medical officers. Dr. Davey then came back to England.

The year after his return Colney Hatch Asylum was opened, and Dr. Davey was appointed one of its medical officers, remaining there until 1853, when he took over the Asylum at Northwoods, near Bristol, which was established and carried on by the late Dr. Henry Hawes Fox (who had been Physician to the Infirmary from 1816 to 1829), succeeded by his son, the late Dr. (afterwards Rev.) W. C. Fox.

There Dr. Davey lived till 1872, when he came to Bristol and took a house at Redland. He then practised generally as physician, but was principally consulted in cases of mental derangement, to the study of which he had devoted nearly the whole of his professional life.

After the death of his wife in 1886, he finally retired from practice, and lived principally with his daughters; he died at the house of Mrs. Ralph Bernard in Kensington. At his own especial desire he was cremated at Woking on the 24th of February.

Dr. Davey must be described as a practitioner of the old school, and he had the courage of his convictions. He was not afraid to bleed his patient if he thought it advisable, or to give the remedies he considered requisite in doses sufficient to have the definite effect, and he practised according to his decision as to what the right course ought to be, as was the custom before the laity became so wise in medical matters, and such critics and judges of the suitable treatment in each case, as they now profess to be.

Dr. Davey's presence was always welcome at the meetings of the Bristol Medico-Chirurgical Society, for he spoke pleasantly and with well-chosen language, and having a few words to say upon very many of the subjects that were brought forward, he helped to make the meetings more lively; and was it not the case that in those earlier meetings in the old room there was a more genial and less ponderous atmosphere than obtains now in the more formal proceedings in our grand new library? He was a regular attendant, and became President in 1882-83, when he gave the usual inaugural address, containing expressions of his own special views, not altogether in unison with the opinions of the majority of his hearers.

It is somewhat difficult to describe Dr. Davey's ideas on many points connected with medicine and its allied sciences, and it is still more difficult to reconcile one set of his opinions with some others. He was a strict follower of Dr. Elliotson in his belief in animal magnetism, and in the transference of the special senses from their normal organs to other parts of the body during the hypnotic state: he was a firm believer in phrenology and the doctrines of Gall and Spurzheim, not infrequently quoting them in his remarks at our meetings when the discussion of some brain case gave him a suitable opening; and latterly he would maintain that the scientific world was coming round to these same opinions, inasmuch as they now recognised the localisation of function of various parts of the body in particular parts of the brain. He fully recognised the existence of "Moral Insanity" as an actual disease of the brain and, biassed by his phrenological ideas, he was wont to attribute many if not most of the crimes so continually recorded in the daily papers, either to disease or to some peculiarity in shape or size of certain "organs" in the cranial map of Gall and Spurzheim.

At that time Dr. Davey was also a firm believer in spiritualism, and he held, according to his statement, daily communication with departed friends. He went so far as to tell the writer of this notice that he not only had daily communication with his dead son, but had also received written communications from him touching on a question of medical treatment.

He was fond of writing, although he cannot be called a voluminous writer. He sent many contributions to the medical press of former days, and wrote and published many pamphlets on medical and political and medico-legal subjects. He discussed in this way, from his points of view (insanity and phrenology), our right of trying and condemning criminals convicted of wilful murder when they were only suffering from the effects of brain disease. He published *Contributions to Mental Pathology*, and later, an octavo volume on *The Physiology and Pathology of the Ganglionic Nervous System*.

Socially, Dr. Davey was a pleasant and cheerful companion. He much enjoyed the society of his fellow-members, whether of our Society or the Bath and Bristol Branch of the British Medical Association, on the Council of which he sat for many years; and at any of our convivial meetings he seemed to be particularly in his element. On the occasion when he was the President of the Bath and Bristol Branch, in the year 1859, he most hospitably received the Council, many of whom drove over to Northwoods from Bristol and Bath. After showing them the establishment and grounds, he entertained them at a very sumptuous dinner.

